# Hybrid Freeze-Dried Dressings Composed of Epidermal Growth Factor and Recombinant Human-Like Collagen Enhance Cutaneous Wound Healing in Rats

**DOI:** 10.3389/fbioe.2020.00742

**Published:** 2020-07-15

**Authors:** Yating Cheng, Yangfan Li, Shiyi Huang, Fenglin Yu, Yu Bei, Yifan Zhang, Jianzhong Tang, Yadong Huang, Qi Xiang

**Affiliations:** ^1^Institute of Biomedicine and Guangdong Provincial Key Laboratory of Bioengineering Medicine, Jinan University, Guangzhou, China; ^2^Biopharmaceutical R&D Center of Jinan University, Guangzhou, China

**Keywords:** full-thickness skin defects, cell proliferation, angiogenesis, freeze-dried dressing, recombinant human-like collagen, epidermal growth factor

## Abstract

Epidermal growth factor (EGF) is important for promoting skin repair and remodeling. Native collagen is also widely used as a scaffold for skin tissue engineering. The limitations of EGF include easy decomposition or inactivation, whereas native collagen is immunogenic and has poor solubility. Therefore, we constructed a freeze-dried dressing based on the recombinant human-like collagen (RHC) to act as a carrier for EGF (RHC/EGF freeze-dried dressing) and promote skin wound closure. Here, the freeze-dried dressing that combined EGF and RHC significantly enhanced the proliferation, adhesion, and spreading of NIH/3T3 fibroblasts and migration of HaCaT keratinocytes at the wound site. The physicochemical characteristics of the RHC/EGF freeze-dried dressing investigated using scanning electron microscopy, Fourier transform infrared (FTIR) spectroscopy, and differential scanning calorimetry revealed that it was a loose and porous cake that redissolved quickly. The molecular mechanisms involved in cell proliferation and angiogenesis were also assessed. The expression levels of the markers Ki-67, proliferating cell nuclear antigen, vascular endothelial growth factor, and cluster of differentiation 31 were significantly increased after treatment with the RHC/EGF freeze-dried dressing (*P* < 0.01, vs. RHC or EGF alone). This increase indicated that the RHC/EGF freeze-dried dressing significantly accelerated wound closure, re-epithelialization, and the orderly arrangement and deposition of collagen in the Sprague–Dawley rats with full-thickness skin defects. This work describes a significant step toward the development of wound environments conducive to healing, and the RHC/EGF freeze-dried dressing is a potential therapeutic strategy in wound management.

## Introduction

Chronic wounds that are labeled as complex and hard-to-heal, as well as those leading to amputations, are health problems that have devastating consequences for patients, healthcare systems, and societies (Olsson et al., 2019). Consequently, enhancing the wound healing process and increasing efficacy of treatments are a major research focus (Ying et al., 2019). Zhao et al. (2020) prepared physical double-network removable hydrogel adhesives with both increased wound healing efficiency and photothermal antibacterial activities. This was achieved via catechol–Fe ^3+^ coordination with quadruple hydrogen-bonding cross-linked amphiphilic polyester and gelatin (Zhao et al., 2020). The use of wound dressings to protect the wound and provide an optimal environment for wound repair is common practice within burn clinics. Although traditional wound healing dressings have improved significantly, wound healing complications remain a significant healthcare challenge (Aljghami et al., 2019). Various types of wound dressing materials including biomimetic extracellular matrices (ECMs) and biomaterials containing growth factors (GFs) are some of the advancements in tissue engineering and biomaterial sciences that have been widely adopted to accelerate wound healing (Choi et al., 2018; Thönes et al., 2019). Epidermal growth factor (EGF) plays a critical role in initiating and sustaining the different phases of wound healing (Hardwicke et al., 2008). Collagen, the most abundant ECM structural protein, has excellent biocompatibility and degradability. Thus, it is considered to be a top candidate for wound dressing preparations (Evan et al., 2019). We constructed a freeze-dried dressing based on recombinant human-like collagen (RHC) and EGF to generate a multifunctional product that can improve cell–biomaterial interactions and promote wound healing.

Numerous studies have examined the potential of EGF for wound repair. Epidermal growth factor not only promotes the proliferation and migration of keratinocytes, but it also improves collagen construction and stimulates the formation and synthesis of the ECM (Chin et al., 2019). However, the proteolytic environment of the wound is one of the major limitations of using EGF. This is because the delivered proteins are effectively degraded in these environments. To overcome the degradation challenge and improve EGF bioavailability, different drug delivery methods have been explored, including sprays, nanoparticle conjugation, and incorporation within dressings. In the future, the main focus will be on developing more patient-friendly and efficient delivery systems through polymer modification and carrier systems (Garg and Goyal, 2014).

Collagen and collagen-based materials have been successfully used in regenerative medicine for over 50 years (Gontijo et al., 2019). Collagen is suitable for tissue engineering because it is a biocompatible and stable structure that forms a large portion of the ECM. In addition, it also provides a physical barrier that is involved in exudate management (Ramanathan et al., 2018). Within the collagen family, type I collagen is widely used as a biological material because it forms fibrils via heterogeneous self-assembly unlike types II and III collagen, which do not (Aljghami et al., 2019). However, natural type I collagen from animal sources can be immunogenic, while at the same time, problems related to the processing techniques, degradation rate, and the disinfection process of type I collagen limit its application. With the development of genetic engineering, a series of RHC, which are highly hydrophilic, safe, and effective, have attracted extensive attention worldwide. Many reports have demonstrated the expression of recombinant truncated type I human-like collagen peptides in *Escherichia coli* (Yang et al., 2004; Guo et al., 2010). In some of these studies, the use of codon optimization allowed for the expression of recombinant type I human-like collagen peptides that contained multiple identical motifs (Yao et al., 2004; Olsen et al., 2005). Some of these motifs are ligands for specific types of integrin receptors. Recombinant type I human-like collagen peptides are not only expected to enhance cell activity through specific integrin-binding receptors but are also extensible and can be used in various fibrous structural materials, including dressings (Grab et al., 1996; Mashiko et al., 2018).

In this study, a new type of RHC was designed and constructed using genetic engineering technology. This RHC contained cell adhesion domains derived from native type I collagen, and this overcame the limitations of native animal-derived collagen. In addition, EGF is capable of controlling biological processes by regulating the proliferation and migration of keratinocytes and epithelial cells. In this study, we sought to determine whether the combination of these two materials would result in a more efficient skin wound dressing. Traditional wound dressings are made of dry gauze, require regular changes, and are prone to secondary injury. In contrast, the ideal characteristics of an improved wound dressing include non-adherence, cost-effectiveness, and the ability to maintain a moist environment for wound healing. To address the limitations described above, we prepared an RHC/EGF (1:1) freeze-dried dressing. Unlike other passive moist wound dressings, the RHC/EGF freeze-dried dressing not only keeps the surrounding wound environment moist but also actively accelerates the wound repair process. Finally, in the model of full-thickness skin defects *in vivo*, RHC/EGF freeze-dried dressings demonstrated strong potential for application in wound healing and repair.

## Materials and Methods

### Reagents

*Escherichia coli* BL21 (DE3) and plasmids containing pET-3c, an ampicillin resistance gene, and isopropyl β-D-1-thiogalactopyranoside (IPTG) were provided by Invitrogen (Carlsbad, CA, United States). Plasmid extraction and gel recovery kits, as well as restriction enzymes and ligase, were purchased from Guangzhou Tianjin Biotechnology Co., Ltd. (Guangzhou, China). Epidermal growth factor was supplied by Jinan University Biopharmaceutical R&D Center (Guangzhou, China). All other reagents were provided by GBCBIO Technologies Inc. (Guangdong, China).

NIH/3T3 (ATCC CRL-7724) cells were purchased from the Chinese Academy of Sciences (Shanghai, China) and cultured in RPMI 1640 supplemented with 10% fetal bovine serum (FBS) (Gibco, Grand Island, NY, United States). HaCaT cells (ATCC CRL-2310) were purchased from the Chinese Academy of Sciences and cultured in Dulbecco modified eagle medium supplemented with 10% FBS. All cell culture plates and bottles were obtained from Corning Company (Corning, NY, United States).

### Construction and Identification of RHC

The gene encoding RHC was cloned into the pET-3c expression vector to generate a recombinant plasmid named pET3c-hlcollagen, which was transformed into *E. coli* BL21 (DE3). After screening for ampicillin resistance and induction by IPTG, the best expression condition was selected. Larger-scale production of RHC was performed using a 50-L fermenter. Moreover, RHC protein was purified using affinity chromatography on a Ni Sepharose 6 Fast Flow column combined with gel filtration Sephadex G-25. Polymerase chain reaction, Western blot, and gel electrophoresis were used for the identification of RHC.

### Interaction Between EGF and RHC

The interaction between EGF and RHC was demonstrated using cell proliferation, migration, and adhesion assays. Proliferation of cells on EGF, RHC, porcine skin collagen, type III collagen, RHC/EGF(0.25:1), RHC/EGF(0.5:1), RHC/EGF(1:1), RHC/EGF(2:1), and RHC/EGF(4:1) was assessed using an MTT assay. Absorbance for each cell culture plate (at 570 nm) was measured in a microplate reader (MK3; Thermo, Waltham, MA, United States). Each assay was performed in triplicate. An *in vitro* scratch wound-healing model was used to assess cell migration. Images of the cells at the beginning of migration and after regular intervals were captured and used to quantify the cellular migration rate. The crystal violet assay was used to measure cell adhesion activity. Images were then captured with an MF53 microscope (Mshot, Guangzhou, China). The experiment was performed in quintuplicate, and the number of adherent cells was calculated from the average of images captured at five positions per well. To investigate cell lysis, a 1% sodium dodecyl sulfate (SDS) solution was added to the culture plate, and the absorbance was measured using a microplate reader at 570 nm (Yuan et al., 2016).

### Cytoskeleton Staining Assays

Coating of 12-well plates with 1 nmol/mL of EGF, RHC, and RHC/EGF was performed at 4°C (overnight). After washing with phosphate-buffered saline (PBS), the wells were blocked with 1% (wt/vol) bovine serum albumin (BSA) for 30 min. NIH/3T3 cells were seeded in the same plates at 4 × 10^4^ cells per well in FBS-free medium. After 4 h, the cells were washed with PBS and fixed with a 4% paraformaldehyde solution for 10 min. Next, phalloidin (1:200; Solarbio, Beijing, China) was used for staining at 37°C for 30 min in the dark, and the cells were washed with PBS (three times). The cells were stained with DAPI (1:1,000; Beyotime, Beijing, China) at room temperature for 5 min and washed with PBS (three times). LSM 700 confocal laser scanning microscope (Zeiss, Wetzlar, Germany) was used for image acquisition, with excitation wavelengths of 488 and 561 nm. The images were analyzed with ImageJ (National Institutes of Health, United States) to calculate the cell adhesion area.

### Preparation of RHC, EGF, and RHC/EGF Freeze-Dried Dressings

Recombinant human-like collagen, EGF, and RHC/EGF (1:1) were lyophilized separately to obtain the freeze-dried dressings. In short, the configured RHC, EGF, and RHC/EGF solutions were subjected to gradient cooling. This involved placing the solutions at 4°C for 1 h, then at −20°C for 6 h, and finally at −80°C overnight. Next, the frozen RHC, EGF, and RHC/EGF were vacuum freeze-dried at −50°C for 24 h. The freeze-dried dressing was sterilized using cobalt-60 gamma irradiation and stored in a sterile dry-sealed EP tube for future experiments.

### Characterization of the RHC and RHC/EGF Freeze-Dried Dressings

The morphologies and structural properties of the freeze-dried dressings were characterized by scanning electron microscopy (SEM, XL30; Philips, Amsterdam, Netherlands), Fourier transform infrared (FTIR, model 8400; Shimadzu, Kyoto, Japan), and differential scanning calorimetry (DSC; Mettler Toledo, Columbus, OH, United States). The micromorphology of RHC and the freeze-dried dressings were observed using SEM after applying gold sputtering on samples using a gold spray carbonator. Fourier transform infrared spectroscopy measurements (in attenuated total reflectance mode) were carried out using a Nicolet iS50 FTIR Spectrometer (Thermo Scientific, Waltham, MA, United States) at room temperature. All the film samples were obtained using the conventional KBr disk method. The samples were scanned in the 400 and 4,000 cm^–1^ range. Thermal curves of RHC, EGF, and RHC/EGF, as well as a physical mixture of RHC and EGF, were obtained using DSC (Mettler Toledo).

### The Rehydration Time of RHC and RHC/EGF Freeze-Dried Dressings

The resolubility tests of RHC and RHC/EGF freeze-dried dressings were performed using physiological saline. In short, we dissolved RHC and RHC/EGF freeze-dried dressings in 300 μL of physiological saline and determined whether the liquid could be sufficiently dissolved (by the degree of liquid clarification) and recorded the dissolution time. All these physical measurements were made in triplicate at 25°C.

### *In vivo* Studies in Full-Thickness Skin Defect SD Rat Model

All SD rats (60 days postnatal) used in this study were purchased from the Animal Centre of Guangdong Province (no. 44007200069979) and caged in a suitable and controlled environment. The experimental protocols used in this study were approved by the Institutional Animal Care and Use Committee of Jinan University (approval 2019228). All experiments were conducted according to the guidelines for animal care and use of China, and the animal ethics committee of the Chinese Academy of Medical Sciences approved them. A full-thickness skin defect model was established to evaluate the role of the RHC, EGF, and RHC/EGF freeze-dried dressings in wound healing. Female SD rats, 200 ± 20 g (*n* = 20 per group) were assigned randomly to three groups: (1) EGF, (2) RHC, and (3) RHC/EGF. Briefly, rats were anesthetized with an intraperitoneal injection of chloral hydrate (10%), and their backs were shaved and cleaned with ethanol. A whole-skin defect model was prepared with a 1.8-cm-diameter ring drill to compress a circular mark on both sides of the rat’s back (2.54 cm^2^), and then the skin was cut off along the mark. One of the wounds was treated with physiological saline as the control group, and the experimental groups on the other side were treated with EGF, RHC, or RHC/EGF freeze-dried dressings, respectively. Postoperatively, antibiotics were administered for 3 days to prevent wound infection. After the respective treatments, the wounds were covered with gauze, which was fixed in place with a sterile bandage. On days 3, 7, 14, and 21, five mice were randomly selected from each group, anesthetized, and sacrificed.

### Macroscopic Evaluation of Wounded Tissues

To measure percent wound closure, the wounds were photographed at 0, 3, 7, 10, 14, and 21 days after wounding and analyzed by ImageJ software. The limits of grossly evident epithelialization were used to measure the wound area. The percent wound closure at each time point was calculated using the following formula: [((initial wound size – current wound size)/initial wound size) × 100].

### Microscopic Evaluation of Hematoxylin and Eosin–Stained Skin Samples

The rats were sacrificed on days 3, 7, 14, and 21 after surgery, and wound tissue was collected. The sample was fixed in 4% neutral buffered paraformaldehyde, dehydrated with ethanol, and exposed to xylene. Then the samples were embedded in paraffin, sectioned at a thickness of 8.0 μm, and placed onto glass slides. Finally, they were examined with hematoxylin and eosin (H&E) as well as Masson trichrome staining. Sections were analyzed, and images were captured by microscopy (Olympus IX71, Tokyo, Japan). Hematoxylin and eosin–stained samples were used to measure the epithelial thickness at 14 and 21 days after wounding.

### Immunohistochemical Analysis

On days 3 and 14, wound tissue specimens harvested from the rat model were used for immunohistochemical (IHC) evaluation. Immunohistochemical staining of vascular endothelial growth factor (VEGF) and cluster of differentiation 31 (CD31) was performed using the streptavidin–biotin method. In brief, sections were dewaxed and microwaved for 10 min to retrieve antigens, and then endogenous peroxidase was blocked by incubation in hydrogen peroxide (3%) for 30 min in the dark. Sections were permeabilized with Triton X-100 (1%) solution and blocked in BSA (5%). Next, sections were incubated with antibodies against VEGF (1:300 dilution, GB14165; Google Biotechnology, Wuhan, China) and CD31 (1:200 dilution, GB11063-3; Google Biotechnology) overnight. After rinsing with PBS, the sections were incubated with rabbit secondary antibody for 40 min. After washing four times with PBS, the DAB and hematoxylin were applied for coloration and redyeing the nucleus, respectively. Finally, sections were dehydrated and sealed with Permount^TM^ Mounting Medium for microscopic observation (Olympus IX71).

### Immunofluorescence Analysis

On days 7 and 14, wound tissue specimens harvested from the rat model were used for IHC fluorescence evaluation. To visualize immunofluorescence, sections were incubated with antibodies against Ki-67 (1:300 dilution, GB13030-2; Google Biotechnology) and proliferating cell nuclear antigen (PCNA, 1:200 dilution, GB11010; Google Biotechnology) overnight. After rinsing with PBS, sections were incubated with a fluorescent rabbit secondary antibody for 40 min. After washing four times with PBS, cell nuclei were stained with DAPI, and images were acquired with a confocal laser scanning microscope (Olympus LSM 700).

### Statistical Analysis

All data were expressed as the mean ± standard deviation (SD) of at least three independent experiments and were analyzed using one-way analysis of variance with a Tukey honestly significant difference comparison test, where *P* < 0.05 was considered statistically significant. GraphPad Prism 6 software (GraphPad Software Inc., La Jolla, CA, United States) was used for statistical analysis.

## Results

### Construction and Identification of RHC

**FIGURE 1:**
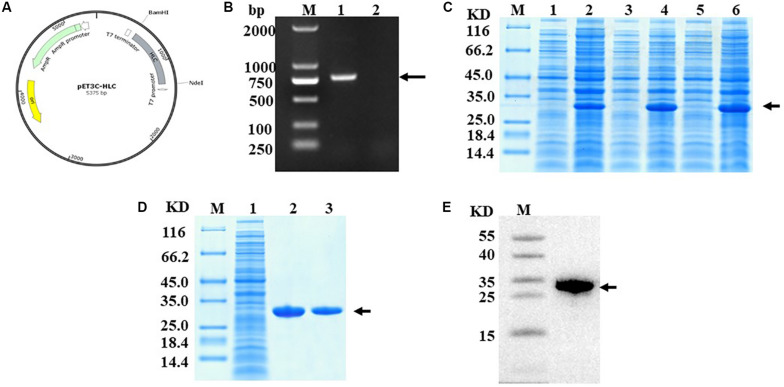
Construction and identification of RHC. **(A)** Construction schematic of the recombinant pET3c-hlcollagen plasmid. **(B)** Nucleic acid electrophoresis of recombinant plasmid pET3C-HLC. M: DNA Ladder 2000; lane 1: monoclone of recombinant plasmid pET3C-HLC; lane 2: negative control. **(C)** Effect of temperature on RHC expression, as analyzed by SDS-PAGE. Recombinant human-like collagen was induced by IPTG for 4 h at either 37 or 30°C or overnight at 20°C. M: middle molecular weight protein markers; lanes 2, 4, and 6: RHC after induction at 20, 30, or 37°C; lanes 1, 3, and 5: RHC before induction. **(D)** Sodium dodecyl sulfate–PAGE analysis of proteins during purification. M: middle molecular weight protein markers; lane 1: broken by PBS; lane 2: Ni-NTA spin columns; lane 3: washed protein through 80 mM imidazole. **(E)** Western blotting analysis. M: middle molecular weight protein markers; lane 1: RHC with an anti-His antibody.

Using genetic engineering technology, we constructed RHC according to the construction schematic shown for the recombinant plasmid of pET3c-hlcollagen (Figure 1A). The results from nucleic acid electrophoresis showed that the RHC gene was successfully ligated into the expression vector (Figure 1B). After induction of the *E. coli* BL21 that contained recombinant plasmid RHC with IPTG (at either 30°C or 37°C) for 4 h, or 20°C overnight, soluble RHC expression was highest at 37°C (Figure 1C). Subsequently, the RHC protein was purified using affinity chromatography on a Ni Sepharose 6 Fast Flow column combined with gel filtration Sephadex G-25. The purity of RHC was detected by SDS–polyacrylamide gel electrophoresis (PAGE) gel electrophoresis (Figure 1D) and Western blot (Figure 1E), and in both of them, a single band was obtained. Identity was confirmed by matrix-assisted laser desorption/ionization–time of flight mass spectrometry and High-performance liquid chromatography–based peptide mapping (data not shown). We conducted at least three consecutive batches of 50-L fermentation process tests, and the mean of RHC protein expression accounted for 48% of the total protein in the bacterial cell supernatant (data not shown).

### Combined RHC With EGF Promotes Proliferation and Migration Synergistically

The proliferation of NIH/3T3 cells cultured on RHC, EGF, porcine skin collagen, type III collagen, and RHC/EGF substrates was assessed. These experiments demonstrated that RHC, porcine skin collagen, and type III collagen did not promote the proliferation of NIH/3T3 cells (Supplementary Figure 1A).

**FIGURE 2:**
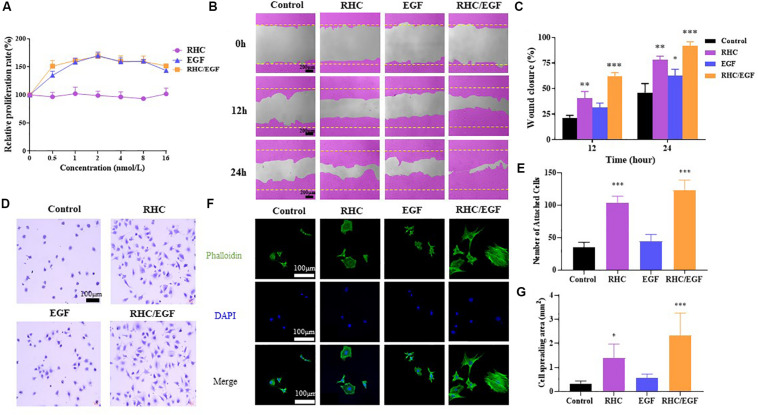
Cell biological activity of RHC/EGF (1:1). **(A)** MTT assay of NIH/3T3 cell proliferation rates on RHC, EGF, or RHC/EGF. **(B)** Images obtained at 0, 12, and 24 h after wound creation *in vitro* migration assay on RHC, EGF, or RHC/EGF. **(C)** Quantitative analysis of gap area of HaCaT cells cultured on RHC, EGF, or RHC/EGF. **(D)** Optical micrographs of crystal violet–stained NIH/3T3 cells adhering to monolayers. **(E)** Quantitative detection of the number of adherent NIH/3T3 cells. **(F)** Cytoskeleton staining. **(G)** Quantitative calculation of cell spread area. *n* = 3, means ± SD, ^∗^*P* < 0.05, ^∗∗^*P* < 0.01, ^∗∗∗^*P* < 0.001 vs. control group.

When the cells were cultured on substrates containing various freeze-dried combinations of RHC and EGF (ratio of RHC/EGF = 0.25:1, 0.5:1, 1:1, 2:1, and 4:1), our results showed that the RHC/EGF (1:1) freeze-dried dressing had the strongest effect on proliferation (Supplementary Figure 3). In addition, EGF also significantly promoted cell proliferation (Figure 2A).

HaCaT cells are considered to be a good *in vitro* model of the skin epidermal layer and can be used to assess the therapeutic effects of different compounds on tissue regeneration. The *in vitro* scratch wound healing assay using HaCaT cells shows that, in the RHC and RHC/EGF group, wound closure by cell migration in HaCaT cells is significantly enhanced when compared with those of untreated or EGF groups (Figure 2B). After a 12- or 24-h incubation with HaCaT cells, markedly higher percent wound closures were observed. The migration characteristics of HaCaT cells on RHC, EGF, and RHC/EGF were examined by the scratch wound assay (Figure 2B). After a 24-h incubation, a significantly higher (*P* < 0.01) percent wound closure is observed in RHC (78% ± 3.12%) and RHC/EGF (91.6% ± 1.62%) compared with that in control (46.3% ± 1.82%) and EGF (62.3% ± 2.12%) (Figure 2C).

### The Joint Application of RHC and EGF Promotes NIH/3T3 Cell Adhesion and Spreading

To investigate the effect of RHC, EGF, porcine skin collagen, type III collagen, or RHC/EGF on cell adhesion, NIH/3T3 cells were cultured in serum-free medium and plated on 96-well plates coated with RHC, EGF, or RHC/EGF for 2 h. The cell adhesion activity of the NIH/3T3 cells was then evaluated using the crystal violet assay. Recombinant human-like collagen and RHC/EGF groups significantly promoted cell adhesion compared with the control and EGF groups (Figure 2D). In addition, compared with porcine skin and type III collagen, the RHC group significantly enhanced cell adhesion (*P* < 0.001, Supplementary Figure 1B). Among all the groups, RHC/EGF had the highest number of attached cells that were well-spread and exhibited a typical fibroblast cell morphology. We also determined the average number of cells attached to monolayers representing the control, RHC, EGF, and RHC/EGF groups (Figure 2E). The largest number of cells was attached to RHC/EGF, with approximately 130 cells attached per field of view. When compared with the control and EGF, cell attachment was significantly lower at 20–30 cells per field.

To further analyze the adhesion activity, we assessed NIH/3T3 cell adhesion to RHC/EGF. Cells were allowed to adhere for 4 h to each of the monolayer substrates, and then samples were fixed and stained to allow for the comparison of cytoskeletal development (Figure 2F). We observed that cells on the RHC/EGF surfaces displayed an organized actin cytoskeleton. In contrast, cells on the control and EGF surfaces lacked an organized actin structure. To quantify the spread of the attached cells, we calculated the cell area using ImageJ (Figure 2G). The results showed that the spread area of the NIH/3T3 cells cultured on the RHC/EGF substrates was significantly larger than the spread area of cells cultured on the control and EGF substrates. These results indicate that compared with the other groups tested, RHC/EGF has the best NIH/3T3 cell adhesion activity.

### Characterization of the RHC/EGF Freeze-Dried Dressings

**FIGURE 3:**
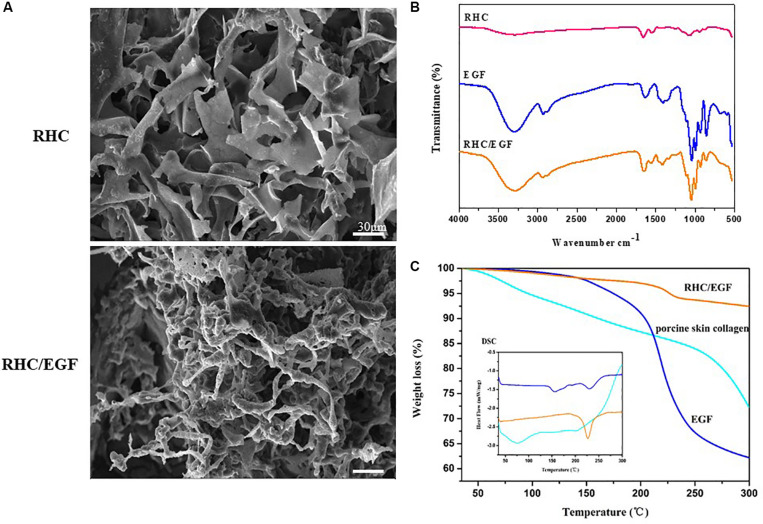
Characteristics of RHC and RHC/EGF. **(A)** Scanning electron micrographs of RHC and RHC/EGF freeze-dried dressings. **(B)** Fourier transform infrared spectra of RHC, EGF, and RHC/EGF freeze-dried dressings. **(C)** Differential scanning calorimetry spectrum of EGF, porcine skin collagen, RHC/EGF freeze-dried dressing.

Recombinant human-like collagen and RHC/EGF (1:1) freeze-dried dressings have a noticeable but irregular pore structure and self-assemble by lyophilization to form a distinct fibrous structure (Figure 3). In addition, solubility experiments showed that both RHC and RHC/EGF freeze-dried dressings could be completely dissolved in physiological saline within a short period (3–5 s), to form a transparent and uniform aqueous solution.

The structural characteristics of the RHC/EGF freeze-dried dressing were examined using FTIR spectroscopy (Figure 3B). The presence of the characteristic bands of collagen in RHC, porcine skin collagen, type III collagen, and RHC/EGF freeze-dried dressings at approximately 1,640, 1,535, and 1,260 cm^–1^ is attributed to the absorption bands of amide I, II, and III, respectively, and exhibits with a hydrogen-bonded amine group (Figure 3B and Supplementary Figure 2A). The carbonyl group with the absorption peak ranging from 1,700 to 1,600 cm^–1^ reveals the presence of a main structural protein in the RHC/EGF freeze-dried dressing. The peak ranging from 3,600 to 3,000 cm^–1^ represents the stretching vibration of -NH bonds. There was no difference seen in the FTIR spectra of these three groups.

The amino acid denaturation temperature of collagen dressings is an essential parameter to consider for biomaterials used in tissue engineering applications. The results of both the DSC and TG (thermogravimetric analysis) indicated that the denaturation temperature of porcine skin and type III collagen was approximately 78 and 73°C, respectively (Figure 3C and Supplementary Figure 2B). The denaturation temperature of EGF was 157.6°C, whereas the denaturation temperature of the RHC/EGF freeze-dried dressing was 224.2°C, which demonstrated that RHC combined with EGF had improved thermal stability. The RHC/EGF freeze-dried dressing had a weight loss of 6.3% in the transition temperature range of 150–250°C compared to 27.3 and 35.6% for native collagen and EGF, respectively (Figure 3C). This result indicates that EGF had a strong interaction with RHC, which increased the thermal stability of RHC/EGF freeze-dried dressings.

### The RHC/EGF Freeze-Dried Dressings Improve Wound Healing of Sprague–Dawley Rats With Full-Thickness Skin Defects

**FIGURE 4:**
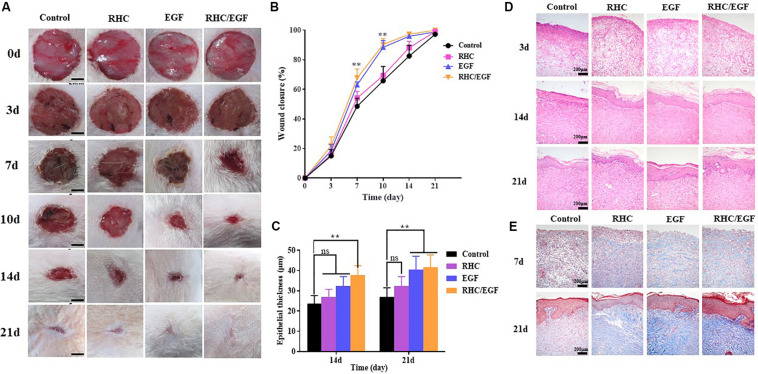
Experimental study of rat wound-healing model after treatment with physiological saline, RHC, EGF, or RHC/EGF. **(A)** Wounds were photographed at days 0, 3, 5, 7, 10, 14, and 21. **(B)** Quantitative analysis of epithelial thickness at days 14 and 21 using ImageJ software. **(C)** Hematoxylin and eosin staining of rat wound-healing model after treatment for 3, 14, and 21 days. **(D)** Quantitative analysis of wound closure rate. **(E)** Masson trichrome staining of rat wound-healing model after treatment for 7 and 21 days. *n* = 5, means ± SD, **P < 0.01 vs. control group, ns means no significant difference vs. control group, *P* > 0.05.

After establishing the full-thickness skin defects model, the wounds were treated with EGF, RHC, and RHC/EGF freeze-dried dressings. Wounds treated with EGF served as the positive control group, whereas physiological saline was administered to the negative control group. Wound closure at each time point for all the experimental groups was measured. The healing time of the RHC/EGF group was reduced compared with that of the other groups, and the wound closure rate of the RHC/EGF group was much higher than that of the other two groups (Figure 4A). At 10 days after wounding, a significantly higher (*P* < 0.01) percent wound closure is observed in the EGF (88.39% ± 4.13%) and RHC/EGF (90.33% ± 7.47%) groups compared with the RHC (69.32% ± 6.18%) and control groups (65.79% ± 9.48%) (Figure 4B). The appearances of the wounds indicated that RHC/EGF freeze-dried dressings improved wound healing, whereas non-treatment resulted in noticeable large and elongated scars.

Next, a histological analysis (via H&E staining) was performed to assess the quality of the newly formed skin tissue (Figure 4C). Hematoxylin and eosin staining results indicated that on day 3, there was a decrease in wound inflammation and significant capillary growth in the RHC/EGF freeze-dried dressing group. In the control group, a large number of inflammatory cells infiltrated the upper layer of the dermis, but there was negligible capillary growth. Evaluation of the skin-injury model after treatment for 14 or 21 days (Figure 4D) indicates a significant increase in epithelial thickness in the RHC/EGF group (41.66 ± 4.62 μM, *P* < 0.01) compared with the control (26.83 ± 3.58 μM) and RHC group (32.33 ± 3.18 μM).

### The RHC/EGF Freeze-Dried Dressing Promotes Skin Collagen Regeneration and Orderly Arrangement

Masson trichrome staining can be used to assess the overall quality of the scar area by measuring collagen deposition and morphology. Masson trichrome staining results showed that the wound treated with RHC exhibited deposition of ECM on day 7, especially for blue-stained collagen (Figure 4E). In addition, at day 21, wound surfaces treated with RHC/EGF had enhanced collagen deposition, as well as highly compact and arranged collagen fibers compared with those in the control group.

### The RHC/EGF Freeze-Dried Dressing Accelerates Both Cell Proliferation and Neovascularization in Skin Defects

**FIGURE 5:**
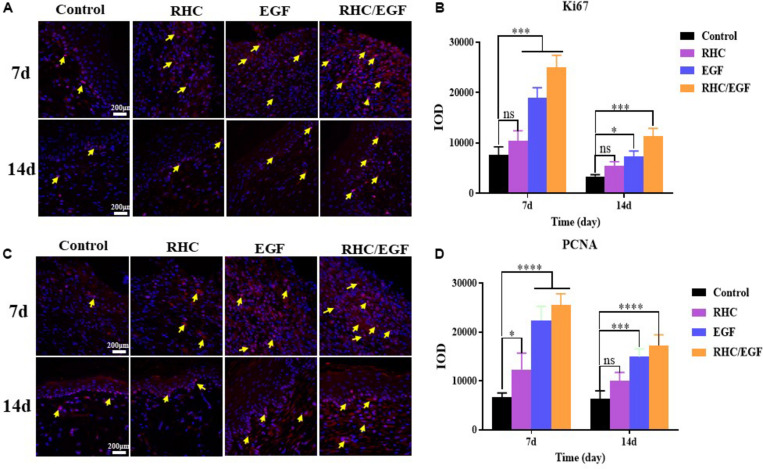
Immunofluorescence examination of Ki-67 and PCNA expression. **(A)** Immunofluorescence examination of Ki-67–positive cells after treatment for 7 and 14 days. Arrows indicate Ki-67 expression. **(B)** Quantitative analysis of Ki-67–expressing cells at 7 and 14 days measured using ImageJ software. **(C)** Immunofluorescence examination of PCNA-positive cells after treatment for 7 and 14 days. Arrows indicate PCNA expression. **(D)** Quantitative analysis of PCNA-expressing cells at 7 and 14 days measured using ImageJ software. *n* = 3, means ± SD, ^*^P < 0.05, ^***^P < 0.001, ^****^P < 0.0001 vs. control group, ns means no significant difference *vs.* control group, *P* > 0.05.

**FIGURE 6:**
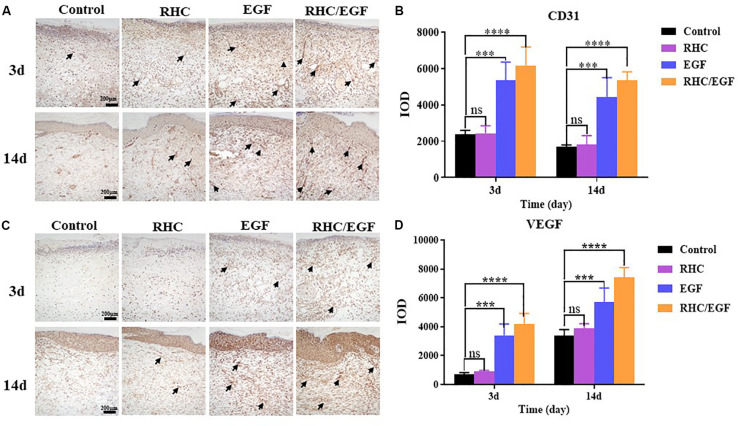
Immunohistochemical examination of CD31 and VEGF expression. **(A)** Immunohistochemical labeling of CD31-positive cells after treatment for 3 and 14 days. Arrows indicate CD31 expression. **(B)** Quantitative analysis of CD31-expressing cells at 3 and 14 days measured using ImageJ software. **(C)** Immunohistochemical labeling of VEGF-positive cells after treatment for 3 and 14 days. Arrows indicate VEGF expression. **(D)** Quantitative analysis of VEGF-expressing cells measured at 3 and 14 days using ImageJ software. *n* = 3, means ± SD, ***P < 0.001, ****P < 0.0001 vs. control group, ns means no significant difference *vs.* control group, *P* > 0.05.

Ki-67 and PCNA are important protein biomarkers for measuring cell proliferation. On days 7 and 14, damaged skin tissues were collected to examine the expression of two cell proliferation–specific proteins, Ki-67 and PCNA (Figure 5). After treatment with RHC/EGF for 7 days, there is a significant increase in the expression of Ki-67 and PCNA (Figures 5A,C). Statistical results showed that there is a significant difference between the RHC/EGF group and the control group (Figures 5B,D; *P* < 0.001). On day 14, the expression of Ki-67 and PCNA is significantly decreased in all groups. Neovascularization is required for normal tissue development, and both CD31 and VEGF are widely used as markers of angiogenesis. Damaged skin was collected on days 3 and 14, and the expression of CD31 and VEGF in the vascular network was assessed by IHC (Figure 6). After treatment for 3 or 14 days, the expression of CD31 is significantly increased in the EGF and RHC/EGF groups compared with the control and RHC groups. Vascular endothelial growth factor expression also exhibited a similar trend at the same time points.

## Discussion and Conclusion

The wound healing process is a dynamic and relatively lengthy process. At present, various interventions, including external dressings, biotherapy, physical therapy, and surgical skin grafts, are used for clinical treatment. Numerous research studies have focused on the development of dressings for wound repair. Zhao et al. (2017) developed a series of injectable and conductive self-healed hydrogels. These were based on the quaternized chitosan-*g*-polyaniline (QCSP), and the benzaldehyde group–functionalized poly(ethylene glycol)-co-poly(glycerol sebacate) (PEGS-FA) used as self-healing wound dressings, which significantly promoted the *in vivo* wound healing process (Zhao et al., 2017). Qu et al. (2018) prepared a series of hydrogels by mixing quaternized chitosan (QCS) and benzaldehyde-terminated Pluronic^®^F127 (PF127-CHO) under physiological conditions as a wound dressing for joint skin wound healing, which were biocompatible and promoted efficient hemostasis (Qu et al., 2018). Wound dressings based on collagen are practical and easily remodeled because of their simple structure, relative uniformity, and abundance. Various wound healing products developed using native collagen (with or without other bioactive compounds) have been available on the market for many years. However, the application of native collagen is limited because of its poor solubility and immunogenicity. Native collagen consists of multiple molecular weight components, and this affects wound dressing consistency and quality. With the improvements in recombinant protein techniques, the research on high-quality and contaminant-free RHC has been growing by leaps and bounds. The RHC may produce a suitable biological dressing for burn wounds as an alternative to native collagen (Shilo et al., 2013; Udhayakumar et al., 2017; Cheng and Hu, 2019). Rutschmann et al. (2014) coexpressed human type III collagen with virus-derived proline 4-hydroxylase in the *E. coli* expression system to obtain RHC with hydroxylated structure (Rutschmann et al., 2014). Many researchers have successfully attempted to produce recombinant type III human-like collagen peptides (Xu et al., 2011; Zhang et al., 2017; Suarez Muñoz et al., 2018). On the wound surface, type I collagen promotes wound healing by providing structural support for cell attachment and migration. Nevertheless, type I collagen is composed of two different peptide chains, and it is more difficult to recombinantly express it compared with type III collagen. Pawelec et al. (2017) recombinantly expressed type I human-like collagen peptides to serve as a scaffold to promote hMSC differentiation (Pawelec et al., 2017). Tye et al. (2005) constructed recombinant type I human-like collagen with a high affinity for the bone sialoprotein and promoted the combination of bone sialoprotein and hydroxyapatite as a scaffold (Tye et al., 2005). We succeed in constructing an RHC composed of type I collagen-derived cell adhesion domains in the *E. coli* expression system and realized the Modern Scale Production. Because of its safety, reliability, good water solubility, and ease of mass production, it is suitable for use as a tissue-material component in the field of skin repair in the future.

According to the SEM results, the RHC has a nanofibrous structure similar to natural collagen, which can keep the skin lesion moist, as well as retain GFs. The FTIR results indicate that there are no significant structural differences between the RHC and RHC/EGF freeze-dried dressings owing to their similar amino acid or polypeptide composition. This finding does not indicate whether there is a chemical bond between EGF and RHC. With regard to the thermal stability, the results from DSC and TG demonstrated that the RHC and RHC/EGF freeze-dried dressings exhibited higher denaturation temperatures when compared with porcine skin collagen or EGF. We designed the RHC/EGF freeze-dried dressing based on the assumptions that (1) RHC has binding sites for fibroblasts and has a chemotactic effect on these cells; (2) EGF promotes proliferation and migration of keratinocytes; and (3) the combination of RHC/EGF has synergistic effects on the process of skin repair and skin remodeling. The feasibility and efficiency were verified by experiments both *in vivo* and *in vitro*. After treating NIH/3T3 cells (fibroblast-like cell) for 48 h, both EGF alone (69.76% ± 5.14%, *P* < 0.01, vs. control group) and together with RHC (68.39% ± 4.55%) significantly enhanced fibroblast cell proliferation compared with RHC alone, suggesting that RHC alone only has the ability to improve adsorption.

In addition, the combination of RHC and EGF accelerates the migration of keratinocytes and significantly promotes the adhesion of NIH/3T3 cells (Figure 2B, *P* < 0.01). The cells spread well, and the organized actin cytoskeleton is found throughout the entire cell (Figures 2D,F). The results of both the scratch wound assay and immunofluorescence staining showed that the RHC/EGF freeze-dried dressing could promote the migration of cells into the wound area. Recombinant human-like collagen acts as a scaffold to retain EGF and facilitate its long-term function. Recombinant human-like collagen and EGF are mutually interdependent and interact to achieve a wide variety of wound repair functions.

To demonstrate the effects of our RHC/EGF freeze-dried dressing on wound healing *in vivo*, a full-thickness rat skin excisional model was used. In the early process of wound healing, tissue defects are gradually filled with migrating and proliferating fibroblasts, keratinocytes, vascular endothelial cells, and macrophages (Rippa et al., 2019). Granulation tissue formation marks the completion of early wound repair, and its rate of formation is directly related to the rate of wound healing. At 7 days after wounding, there are significant differences between the groups. At 10 days after wounding, the percent wound closure of the RHC/EGF freeze-dried dressing group is greater than 90% (*P* < 0.01), and the wound is generally healed (Figure 4B). This phenomenon can be explained by the fact that RHC/EGF promotes the proliferation of fibroblasts and the formation of tiny blood vessels, thus accelerating the formation of granulation tissue and shortening the time for wound healing. In fact, our experiments confirmed the results. When treated with the RHC/EGF freeze-dried dressing, the angiogenic markers CD31 and VEGF in the wound site are significantly upregulated (*P* < 0.01). This indicated to us that the capillary number increased significantly. Wound healing involves the spatiotemporal synchronization of multiple cell types in the hemostasis, inflammation, growth, re-epithelialization, and remodeling phase. The upregulation of cell proliferation biomarkers Ki-67 and PCNA on the third day suggested that the RHC/EGF dressing enhanced cell proliferation in the early stages of wound healing significantly. We observed that the RHC/EGF dressing group healed without scarring, 21 days after wounding. As the healing process continues, regenerative scarless healing greatly depends on the well-controlled reestablishment of normal ECM during the remodeling phase. To assess the quality of the newly formed skin tissue, we used both H&E and Masson trichrome staining. After treatment with the RHC/EGF dressing, histological staining demonstrated that the newly deposited collagen at the wound site had a more orderly arrangement compared with the control group, and the wound area had more collagen fibers and deposition, as well as a significantly increased epithelial thickness.

The primary focus of burn care is the optimization of functional and cosmetic outcomes so that survivors can return to a normal life. Zhao et al. (2018) reported injectable antibacterial conductive cryogels based on carbon nanotubes and glycidyl methacrylate-functionalized quaternized chitosan, which enhanced hemostasis in rabbit liver defects (Zhao et al., 2018). A large number of burns are relatively small and do not affect the quality of life, but even minor burns, if not handled properly, can result in scarring. This can limit function and result in a decreased quality of life. Although many paramedics are skilled at handling small wounds, they are not well-equipped to provide the care that prevents the occurrence of scarring (Greenhalgh, 2016). Epidermal growth factor has a strong effect on wound repair (acute, chronic or large-area burn), and it has also been employed for clinical interventions such as diabetic foot ulcer management (Park et al., 2018). To continually improve the EGF-carrier vector structure and maximize therapeutic effects against pathological processes, we also need to perform rigorous exploration. Based on the results of the full-thickness rat skin excisional model, we speculate that the RHC/EGF dressing can be extensively used in burn and chronic dermal ulcers (such as diabetic ulcers). In addition, whether the RHC/EGF dressing can act as a stem cell attractant is of great interest to us and will form the basis of future studies.

In this study, we constructed an RHC with better cell adhesion properties and also achieved our goal to produce relatively large quantities of RHC. Compared with NIH/3T3 cells cultured on RHC or EGF alone, cells on RHC/EGF demonstrated better adhesion, viability, and migration characteristics (*P* < 0.01). The freeze-dried RHC/EGF dressings are more like the natural collagen fibrous structure when compared with the RHC dressing alone. Consequently, we investigated its potential application in wound healing and repair via full-thickness skin defect SD rats. At 7 days after wounding, the percent wound closure in the RHC/EGF group (67.72% ± 4.91%) was significantly higher than the control groups. At 10 days after wounding, the RHC/EGF-treated group recovered. At 21 days after wounding, the RHC/EGF group healed without scarring, whereas non-treatment developed some scars. The wound treated with the RHC/EGF dressing had more collagen fibers and deposition, and the arrangement was denser and more orderly than the control group. Moreover, the wound treated by RHC/EGF upregulated expression of the angiogenesis biomarkers VEGF and CD31, as well as the cell proliferation–specific proteins Ki-67 and PCNA. Collectively, these results support the therapeutic value of the RHC freeze-dried dressing as a topical biomaterial dressing and EGF carrier for regenerative therapies. Because the RHC/EGF freeze-dried dressing is easy to prepare, can be produced on a large scale, and has a clear mechanism of action, we believe that it has great potential for clinical application in the treatment of skin wounds.

## Data Availability Statement

The datasets presented in this study can be found in online repositories. The names of the repository/repositories and accession number(s) can be found in the article/ Supplementary Material.

## Ethics Statement

The experimental protocols used in this study were approved by the Institutional Animal Care and Use Committee of Jinan University (Approval number: 2019228). All experiments were conducted according to the guidelines for animal care and use of China, and they were approved by the animal ethics committee of the Chinese Academy of Medical Sciences.

## Author Contributions

QX and YH contributed to the study conception and design. YC and YL contributed to the acquisition of data and study conduct. YL, FY, and SH contributed to the analysis of data. YB and JT contributed to the materials. YB, SH, and YZ helped perform the analysis with constructive discussions. YC and YL wrote the manuscript. QX and YH modified the manuscript. All the authors contributed to the article and approved the submitted version.

## Conflict of Interest

The authors declare that the research was conducted in the absence of any commercial or financial relationships that could be construed as a potential conflict of interest.
