# Supplementary material for: Hybrid Freeze-Dried Dressings Composed of Epidermal Growth Factor and Recombinant Human-Like Collagen Enhance Cutaneous Wound Healing in Rats
Source: Front Bioeng Biotechnol. 2020 Jul 15;8:742. doi: 10.3389/fbioe.2020.00742 (PMC7375021; doi:10.3389/fbioe.2020.00742)
Supplement: Supplementary file 1 [file Image_1.pdf]

Supplementary Material

1 Supplementary Figures

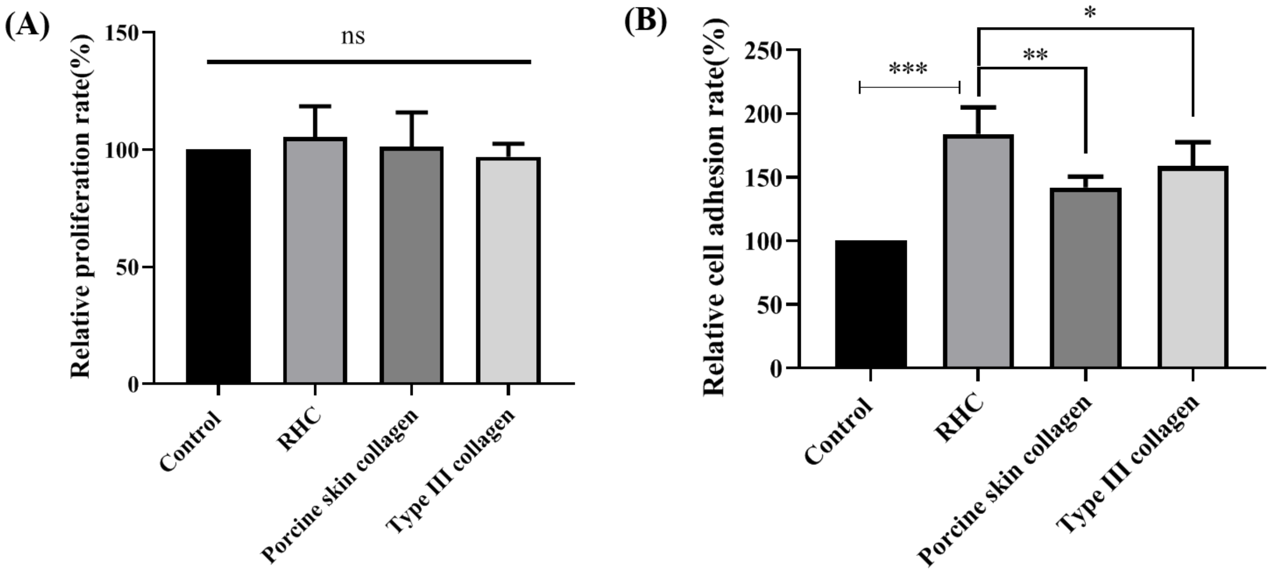

Supplementary Figure 1. Cell biological activity of collagen. (A) MTT assay of NIH/3T3 cell proliferation rates on RHC, porcine skin collagen and type III collagen. (B) cell adhesion rates on RHC, porcine skin collagen and type III collagen. n = 5, means ± SD, *P < 0.05, **P < 0.01, ***P < 0.001 *vs* control group, ns means no significant difference *vs.* control, P >0.05.

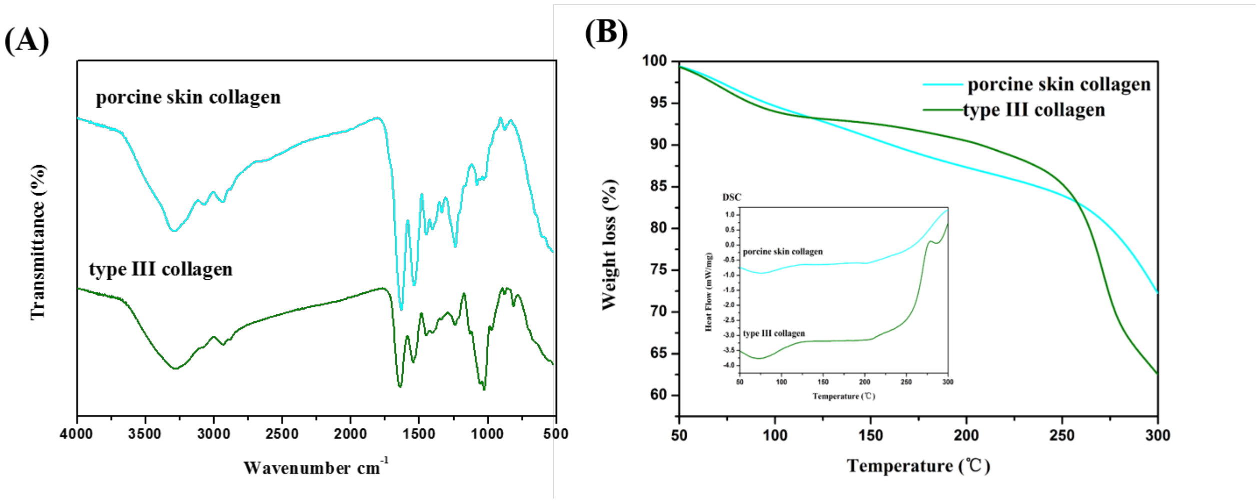

Supplementary Figure 2. Characteristics of porcine skin collagen and type III collagen. (A) FTIR spectra of porcine skin collagen and type III collagen. (B) DSC spectrum of porcine skin collagen and type III collagen .

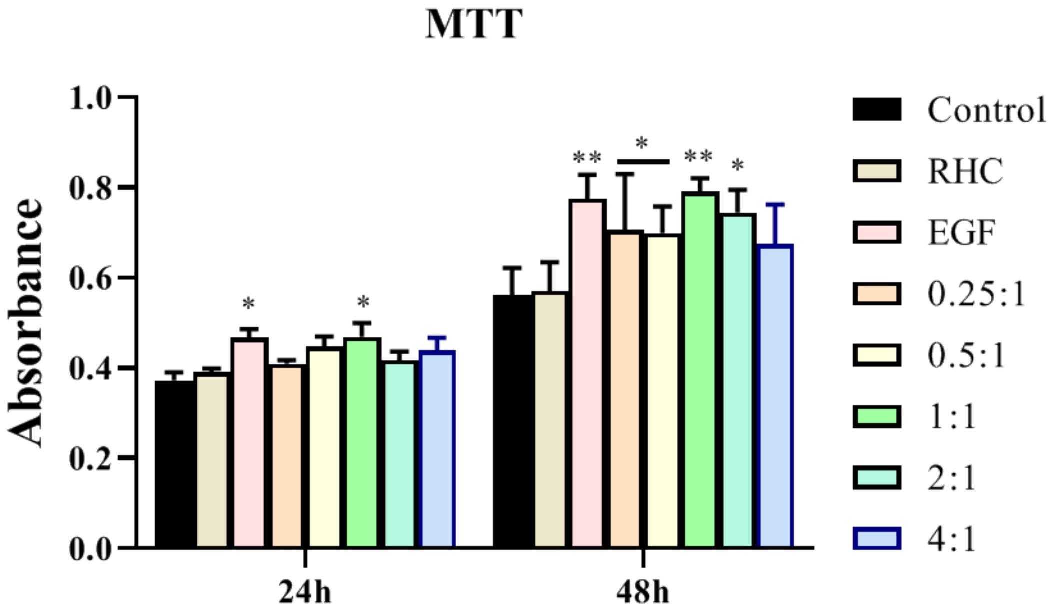

Supplementary Figure 3. MTT assay of NIH/3T3 cell proliferation rates on RHC/EGF(0.25:1), RHC/EGF(0.5:1), RHC/EGF(1:1), RHC/EGF(2:1) and RHC/EGF(4:1). n = 4, means ± SD, *P<0.05, **P<0.01 *vs* control group.
